# mCiRNA—Synaptic Crystal Ball?

**DOI:** 10.18632/aging.100511

**Published:** 2012-12-08

**Authors:** Dale Bredesen

**Affiliations:** The Buck Institute for Research on Aging, Novato, CA 94945

Alzheimer's disease represents a major, and increasing, health care problem, the recognition of which led to the passage of the National Alzheimer's Project Act in 2011 and the creation of the first national plan for Alzheimer's disease (AD). One of the critical needs in AD therapeutic development is for a practical and accurate predictive test that would potentially allow pre-symptomatic treatment. Cerebrospinal fluid abnormalities have been described years prior to symptom development, but regular lumbar punctures represent a sub-optimal screening approach. Amyloid imaging studies offer an alternative, but these are expensive, not yet generally available, and the implications of positive studies in asymptomatic individuals are not yet completely determined. Proteomic analyses of plasma show promise in filling this void [1], and recently, Sheinerman et al. have described a novel and very interesting approach, evaluating the ratios of specific plasma miRNAs [2]. The authors chose 32 neuronal-enriched and brain-enriched miRNAs, especially those associated with neurite and synapse-associated processes, then analyzed each possible ratio in plasma samples, and identified ratios associated with MCI (mild cognitive impairment), AD, and aging. The stability of these miRNAs in plasma allowed for such analysis. The critical ratios identified exhibited sensitivities in the 0.79-0.95 range, with specificities in the 0.79-1.00 range in distinguishing MCI patients from age-matched cognitively normal controls. Interestingly, the same pairs showed smaller differences between normals and patients with AD, and the authors suggested that this may be due to the degeneration associated with AD (and less with MCI), leading to a loss of the synapse and neurite-associated miRNAs that form the basis for the assay, in much the same way that end-stage liver disease is often associated with a more modest increase in “liver function tests” than is earlier, more active liver disease.

Perhaps the most exciting result was that, in a prospective analysis, seven of ten patients who developed MCI displayed predictive miRNA abnormalities from six to 61 months prior to MCI diagnosis. Clearly, more work needs to be done to follow up: for example, did the MCI patients with the normal miRNA ratios revert to normal, continue with MCI, or progress to AD? How early does the ratio become abnormal, given a larger sample size? Are similar results obtained for amnestic MCI and non-amnestic MCI? Single domain vs. multidomain? What about controls with other neurodegenerative diseases—do these show similarly abnormal ratios? However, overall, the results are very promising, suggesting a simple, practical test for neurodegeneration that is capable of detecting abnormalities at a pre-symptomatic stage. Furthermore, some of the questions raised are already being addressed in ongoing follow-up studies.

One critical question relates to the specificity of the test: since the miRNAs are enriched in neurites and synapses, are similar abnormalities detected early in other neurodegenerative diseases such as Lewy body disease or frontotemporal dementia? In other words, is the test described truly a test for MCI and AD, or is it a test for neurodegeneration? Either would of course be welcome, especially if pre-symptomatic patients can be identified, but it will be important to know the test's disease specificity, and how to follow up an abnormal screening result. Another potential issue is the overlap in the control values with those of the MCI and AD patients, suggesting that, on a patient-by-patient basis, the test may be difficult to interpret. It will also be important to generate age-specific normal values, since the group identified age-associated increases.

One of the potentially exciting implications of the study is that MCI may result from different upstream inducers: different miRNA sets that were independently predictive of MCI showed little overlap, arguing for different pathogenetic processes leading to what may currently be lumped as MCI. The ability to classify subgroups of amnestic MCI, for instance, may be valuable in designing and testing specific therapeutics, as well as providing new insights into the underlying mechanistics.

The study by Sheinerman et al. provides an important new analysis in the growing armamentarium that will be critical to the pre-symptomatic and early-symptomatic treatment of neurodegenerative diseases such as Alzheimer's disease.
